# PICK1 Deficiency Induces Autophagy Dysfunction via Lysosomal Impairment and Amplifies Sepsis-Induced Acute Lung Injury

**DOI:** 10.1155/2018/6757368

**Published:** 2018-10-03

**Authors:** Yunchang Mo, Yingying Lou, Anqi Zhang, Jingjing Zhang, Congying Zhu, Bo Zheng, Dan Li, Mingyuan Zhang, Wenjun Jin, Lei Zhang, Junlu Wang

**Affiliations:** ^1^Department of Anesthesiology, The First Affiliated Hospital of Wenzhou Medical University, Nanbaixiang, Wenzhou City, Zhejiang Province 325000, China; ^2^Department of Anesthesiology, The People's Hospital of Wencheng, Wencheng County, Wenzhou City, Zhejiang Province 325300, China

## Abstract

Sepsis is a systemic inflammatory reaction caused by infection. Multiple organ failure ultimately leads to high morbidity and mortality. Unfortunately, therapies against these responses have been unsuccessful due to the insufficient underlying pathophysiological evidence. Protein interacting with C-kinase 1 (PICK1) has received considerable attention because of its important physiological functions in many tissues. However, its role in sepsis-induced acute lung injury (ALI) is unclear. In this study, we used cecal ligation and puncture (CLP) to establish a septic model and found that decreased microtubule-associated protein-1light chain 3 (LC3)-II/LC3-I in PICK1^−/−^ septic mice was caused by autophagy dysfunction. Consistently, the transmission electron microscopy (TEM) of bone marrow-derived macrophages (BMDMs) from PICK1^−/−^ mice showed the accumulation of autophagosomes as well. However, more serious damage was caused by PICK1 deficiency indicating that the disrupted autophagic flux was harmful to sepsis-induced ALI. We also observed that it was the impaired lysosomal function that mediated autophagic flux blockade, and the autophagy progress was relevant to PI3K-Akt-mTOR pathway. These findings will aid in the potential development of PICK1 with novel evidence of autophagy in sepsis treatment and prevention.

## 1. Introduction

Sepsis is a disease closely related to immune function disorders, and severe systemic inflammatory response to infection and complex clinical syndromes associated with sepsis cause death worldwide. Despite advances in treatment, sepsis still remains a life-threatening condition characterized by septic shock and organ failure complications [1]. The lung is always the first organ to be affected by sepsis. Acute lung injury (ALI) and acute respiratory distress syndrome (ARDS) are often the major complications of sepsis and associated with multiple organ failure [2]. As previously reported, the mortality rate of septic lung injury patients is greater than 40%, with tremendous international economic and social burden [3, 4].

Autophagy is one of the innate immune defense mechanisms against microbial challenges caused by serious sepsis [5]. Previous studies have shown that autophagy was induced in lung diseases of septic patients and animals [6]. However, the pathophysiology of these findings has not been elucidated, and whether autophagy plays a protective or harmful role is also not clarified. Autophagy is a fundamental degradation system in cells and involved in establishing an intracellular homeostasis. It is regarded as a second form of programmed cell death distinguished from apoptosis [7]. Autophagy represents an inducible response to stress including hypoxia, cigarette smoke exposure, and inflammation [8]. Dysfunctional and senescent organelles or cytosolic components are enveloped by autophagosomes, followed by transferring to lysosomes for disposal [9]. The basal autophagy serves to degrade aged and defective cellular organelles and macromolecules for reprocessing; however, when the autophagic flux was disrupted, accumulation of damaged proteins or organelles such as mitochondria would further damage the lung tissue. The complete autophagic process is dependent on normal lysosomal function, and inhibition of autophagosome degradation caused by impaired lysosome could induce autophagy dysfunction [10, 11].

Protein interacting with C-kinase 1 (PICK1) harbors a unique structure containing both BAR (Bin/Amphiphysin/Rvs) and PDZ (PSD-95/DlgA/ZO-1) domains, allowing its interaction with various transporters to regulate protein trafficking [12]. PICK1 is abundant in many tissues, especially in the brain and testis, and moderately expressed in the lungs [13]. Wang et al. have reported that PICK1 participates in ROS metabolism and is associated with impaired glutathione synthesis, with PICK1^−/−^ mice showing increased oxidative stress accompanied by subsequent neurodegeneration [14]. Besides, PICK1 influences the progress of acrosome biogenesis, and its deficiency is accompanied by increased apoptosis [15]. PICK1 is also proved to have an anti-inflammatory role in LPS-induced acute liver injury by suppressing macrophage polarization [16]. A recent study showed that the failure of PICK1 localization to nucleus-associated acrosomic vesicles influences acrosome biogenesis in Sirtuin-1-deficient germ cells, and the progress was associated with disrupted autophagic flux [17]. But the relationship between PICK1 and autophagy is not clarified. Based on the previous evidence, we explored the relationship between PICK1 and autophagy progress.

In this study, we explored the function of PICK1 and demonstrated the underlying molecular mechanism on autophagy progress through setting sepsis models in vivo and vitro. Our data demonstrated that PICK1 deficiency caused disruption of autophagic flux and aggravated lung damage induced by sepsis. Additionally, we found that the autophagic flux was halted by impaired lysosomal function. What is more, we studied the role of PI3K-Akt-mTOR pathway in the PICK1-dependent autophagy. The current study developed a more comprehensive role of PICK1, and the dysfunction of autophagy caused by its deficiency is detrimental to sepsis-induced ALI.

## 2. Materials and Methods

### 2.1. Mice

PICK1^−/−^ mice were obtained from Professor Shen Ying (Zhejiang University, Zhejiang, China) [14]. Male wide-type C57BL/6J (6–8-week-old) mice were acquired from the animal center of Wenzhou Medical University (Wenzhou, China). The animals were maintained on standard chow and tap water, in a temperature-controlled chamber at 24°C with a 12 h light–dark cycle. During treatment, the mice were randomly assigned to different groups throughout the study. Experimental protocols were approved by the animal research ethics committee of Wenzhou Medical University. All procedures involving animals were performed in adherence to the Care and Use of Laboratory Animals (Committee for the Update of the Guide for the Care and Use of Laboratory Animals, 2011).

### 2.2. Sepsis Model

A sepsis model was established by CLP as previously described [18, 19]. Under pentobarbital anesthesia administered by ketamine (10 mg/kg body weight, China), a vertical incision (~1 cm) was made at the median of the abdomen to expose the cecum, which was ligated 1 cm to its distal end. The cecum was punctured twice with a 22-gauge needle to extrude a small amount of feces, followed by return of the cecum to the anatomical cavity without retention of any feces outside of the abdomen and suture of the abdominal wall using a 4-0 surgical suture. Mice were postoperatively injected with 1 mL 0.9% warm saline subcutaneously. Food was restricted, but water was freely available in the absence of antibiotics or analgesics. Sham-operated mice were operated on identically without ligation and puncture of the cecum. The animals were killed on a time gradient of 4, 8, and 24 h post-CLP in batches. Lungs were infused with cold saline and reserved for respective evaluation. In the experimental groups, the autophagy inhibitor chloroquine (60 mg/kg body weight; Sigma-Aldrich) was administrated intraperitoneally 1 h after the operation [1, 9]. The control group received an equivalent volume of sterile saline intraperitoneally.

### 2.3. Detection of Total Protein Concentration in BALF

A cervical median incision was made to expose the trachea, and a tracheotomy was performed to harvest BALF. PBS (1 mL) was perfused for each harvest, and BALF acquisition was acquired after repeated for three times. The collected fluid was centrifuged at 500g for 10 min at 4°C, and the supernatant was used for protein detection using a BCA protein assay kit (Thermo Fisher Scientific). Residual fluid was stored at −80°C for cytokine analysis.

### 2.4. Cytokine Analysis

Concentrations of mouse IL-1*β* (VAL601; R&D Systems, Minneapolis, MN, USA) and mouse TNF-*α* (VAL609; R&D Systems) in bronchoalveolar lavage fluid (BALF) were measured using their respective ELISA kits (R&D Systems). Absorbances were recorded using a multifunctional microplate reader (Thermo Fisher Scientific, Waltham, MA, USA).

### 2.5. Detection of Tissue Wet : Dry Weight Ratio

Tissue from the right upper lobe of the lung from each mouse was removed and weighed immediately after removal, and the wet weight was recorded. Lung tissues were desiccated in an oven at 60°C for 2 days until reaching a stable dry weight. The wet : dry weight ratio was calculated accordingly.

### 2.6. Cell Culture and Stimulation

BMDMs were cultured as previously described [20]. BMDMs flushed from mouse femurs and tibias were plated in 6 cm dish with DMEM supplemented with 10% FBS (Sigma-Aldrich) and 20% L929 cell medium a humidified incubator (5% CO_2_). The culture was continued for 7 days to induce the differentiation of harvested cells into macrophages. BMDMs were exposed to LPS (1 *μ*g/mL) and IFN-*γ* (1 U/mL) to establish a septic model.

### 2.7. Histological Analysis

Lungs were perfused with cold saline, followed by administration of 4% paraformaldehyde. After fixation at 4°C for 24 h, tissues were embedded in paraffin, cut into sections (5 *μ*m), and stained with H&E. Lung-injury scores were reviewed in a blinded manner and determined according to four independent parameters: alveolar edema, hemorrhage, infiltration of inflammatory cells, and thickened alveolar septum.

### 2.8. Protein Preparation and Western Blot Analysis

Lung tissues were dissociated using immunoprecipitation assay buffer (Solarbio, Beijing, China) containing protease inhibitors (1 : 100) and phosphatase inhibitors (1 : 50). Extracts were homogenized and centrifuged at 12000g for 30 min at 4°C, followed by storage of the supernatant at −80°C. Protein concentration was measured using a BCA protein assay Kit (Thermo Fisher Scientific) to prepare a system with 1x loading buffer. Protein (50 *μ*g) was loaded per lane and separated by 8% to 12% SDS-PAGE, followed by transfer to a 0.22 *μ*m PVDF membrane (Bio-Rad, Hercules, CA, USA) for 1.5 h. The membranes were subsequently incubated with 5% BSA for 1 h at room temperature to block the nonspecific binding. Primary antibodies were incubated overnight at 4°C, followed by washing of the membranes three times with TBS-Tween 20, and incubation with a horseradish peroxidase-conjugated secondary antibody (1 : 5000) for 1.5 h at room temperature. The following primary antibodies were used: Anti-PICK1 (NeuroMab, Davis, CA, USA), anti-LC3-II, anti-*β*-actin, anti-Akt, anti-p-Akt, anti-mTOR, and anti-p-mTOR were purchased from Cell Signaling Technology (Danvers, MA, USA). Anti-P62, anti-LAMP1, and anti-cathepsin B were purchased from Abcam (Cambridge, UK). Detection was performed using chemiluminescent detection reagent (Thermo Fisher Scientific, Waltham, MA, USA). Band images were scanned using a scanner (Bio-Rad), and image densities were normalized to *β*-actin.

### 2.9. Real-Time RT-PCR

Total RNA was extracted from lung tissue (1 mL/100 mg) and cells (1 mL/well) with Trizol reagent (Life Technologies, Carlsbad, CA, USA). After measuring concentrations of the single-stranded RNA, cDNA was synthesized using random hexamers (Thermo Fisher Scientific) under the following conditions: 5 min at 25°C, 60 min at 42°C, and 5 min at 70°C. The real-time RT-PCR was performed using iTap Universal SYBR Green supermix (Bio-Rad) and the following primers: LC3-II forward (5′-GCAGCTGCCCGTCCTGGACAA-3′) and reverse (5′-TGAGCTGCAAGCGCCGTCTGA-3′); PICK1 forward (5′-AAAGC CATCCCTGATACACG-3′) and PICK1 reverse (5′-TTCCTCGTCATCCATCTCCT-3′); and GAPDH forward (5′-GGTTGTCTCCTGCG AC TTCA-3′) and GAPDH reverse (5′-TGGTC CAGGGTTTCTTACTC C-3).

PCR reactions were performed using the following conditions: 95°C for 3 min, 39 cycles of 95°C for 10 s, and 55°C for 30 s. Reactions were performed on a CFX96 real-Time PCR detection system (Bio-Rad), and quantification results were analyzed with CFX Manager software (Bio-Rad).

### 2.10. Immunofluorescence Microscopy

Lung tissues were cut into sections as mentioned in the histological analysis above. After dewaxed in the oven for 1 h, the sections were put in the xylene and dehydrated in an alcohol gradient for every 5 mins. Then, the sections were retrieved by microwave antigen retrieval with citric acid buffer (pH6.0) for 20 mins. After washing with PBS twice, sections were permeabilized with 0.3% Triton X-100. Nonspecific binding was blocked with 5% donkey serum at room temperature for 1 hour before they were incubated with primary antibodies against LAMP1 (1 : 100) overnight at 4°C. After washing with PBS three times, sections were incubated with fluorescent secondary antibodies DyLight 594 donkey anti-rabbit lgG (H+L) (Jackson, USA) for 1 h at room temperature. Nuclei were stained with DAPI and imaged with a fluorescence microscope (Leica, Wetzlar, Germany). At least four different fields were measured per section and used for quantification with NIH ImageJ. The fluorescence intensity was calculated by the pixels in the region of interest.

### 2.11. Transmission Electron Microscopy (TEM)

BMDMs (1 × 10^7^ cells/well) were harvested and incubated with 2.5% glutaraldehyde at 4°C overnight. Cells were centrifuged and osmicated for 60 min at 4°C, followed by washing with water twice. After dehydration using an alcohol gradient, cells were embedded in Epon 812 at room temperature for 2 h. Cells were cured at 70°C overnight, followed by sectioning with an ultramicrotome and staining with uranyl acetate and Reynold's lead citrate. Autophagosomes and autophagosome–lysosome complexes were examined using a transmission electron microscope (TEMCNAI-10; Philips; FEI Company, Hillsboro, OR, USA). Each cell group had three independent experiment, and each section was observed randomly for 10 fields under 10,500x magnification.

### 2.12. Statistical Analysis

Data are represented as the mean ± SEM and were analyzed using the GraphPad Prism 5.0 software (GraphPad Inc., San Diego, CA, USA). Statistical analysis was performed using the one-way ANOVA and two-way ANOVA followed by the Student-Newman-Keuls test. A *P* < 0.05 was considered statistically significant.

## 3. Results

### 3.1. PICK1 Was Increased with Activated Autophagy Progression in Sepsis-Induced ALI

**Figure 1:**
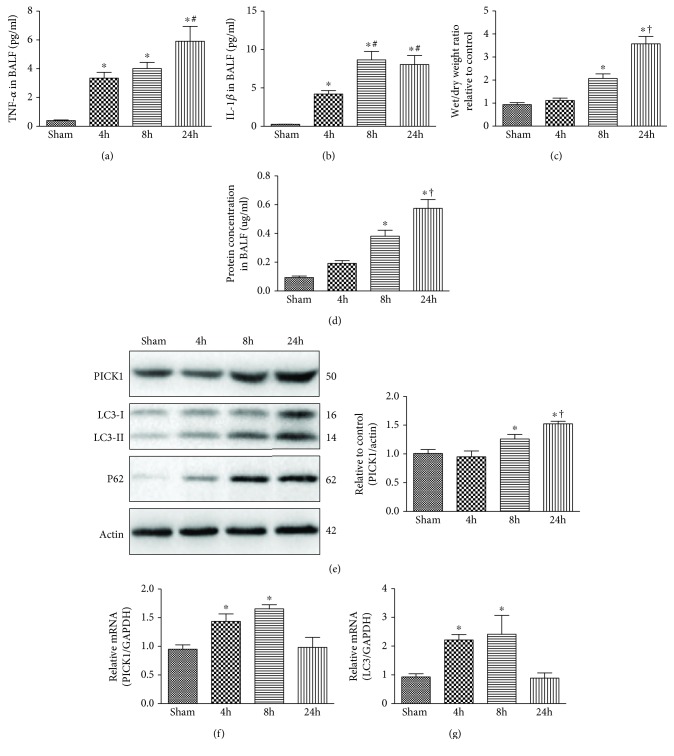
PICK1 participates in autophagic progression in sepsis-induced ALI. (a, b) TNF-*α* and IL-1*β* concentrations were measured in BALF. (c, d) Lung injury was evaluated by lung wet : dry weight ratio, and permeability was assessed by detecting changes in total BALF protein concentration. (e) Lungs were harvested at 4 h, 8 h, and 24 h post-CLP, and Western blot was performed to analyze PICK1, LC3-II/LC3-I, and p62 content. Representative Western blot images and the statistical density of PICK1 are shown in the graphs. (f, g) PICK1 and LC3 mRNA levels measured in the lungs by quantitative PCR. Results represent the mean ± SEM (*n* = 8/group). ^∗^*P* < 0.05 vs. sham-operated groups; ^#^*P* < 0.05 vs. 4 h post-CLP; ^†^*P* < 0.05 vs. 8 h post-CLP.

To investigate the importance of PICK1 in autophagy regulation, we established sepsis models for observation at 4 h, 8 h, and 24 h. Firstly, we measured cytokine and tissue-edema levels to evaluate the degree of lung injury. Both TNF-*α* and interleukin (IL)-1*β* in BALF significantly increased at 4 h and maintained this high level at 24 h (Figures 1(a) and 1(b)). The wet/dry weight ratio and the protein concentration in BALF also increased at 8 h, reaching a peak at 24 h (Figures 1(c) and 1(d)). However, there was no difference in the sham group killed at different time (data were not shown). These results indicated a success of sepsis model for septic model and excluded the deviation caused by operation on the sham mice. A marker of autophagy activation includes transformation from microtubule-associated protein-1 light chain 3 (LC3-I) to LC3-phosphatidylethanolamine conjugate (LC3-II). Additionally, p62, a selective substrate for autophagy, is also an indicator of autophagic flux [21, 22]. In order to monitor the autophagy progress, the conversion of LC3-I to LC3-II and p62 was measured by Western blot assay. We observed that LC3-II/LC3-I and p62 levels were slightly increased at 4 h and maintained a sustainable increase at 8 h and 24 h as compared with sham-operated mice (Figure 1(e)). Interestingly, PICK1 protein levels stayed unchanged at 4 h but progressively increased at 8 h and 24 h after CLP. What is more, PICK1 mRNA expression changed consistently with LC3 levels, with both increasing at 4 h and 8 h post-CLP before returning to baseline levels at 24 h as compared with sham-operated mice (Figures 1(f) and 1(g)). As previous research has confirmed, autophagy was activated at the early stage of sepsis [1]; we speculate that PICK1 participates in autophagy progression related to sepsis-induced ALI.

### 3.2. PICK1 Deficiency Blocked Autophagic Flux

**Figure 2:**
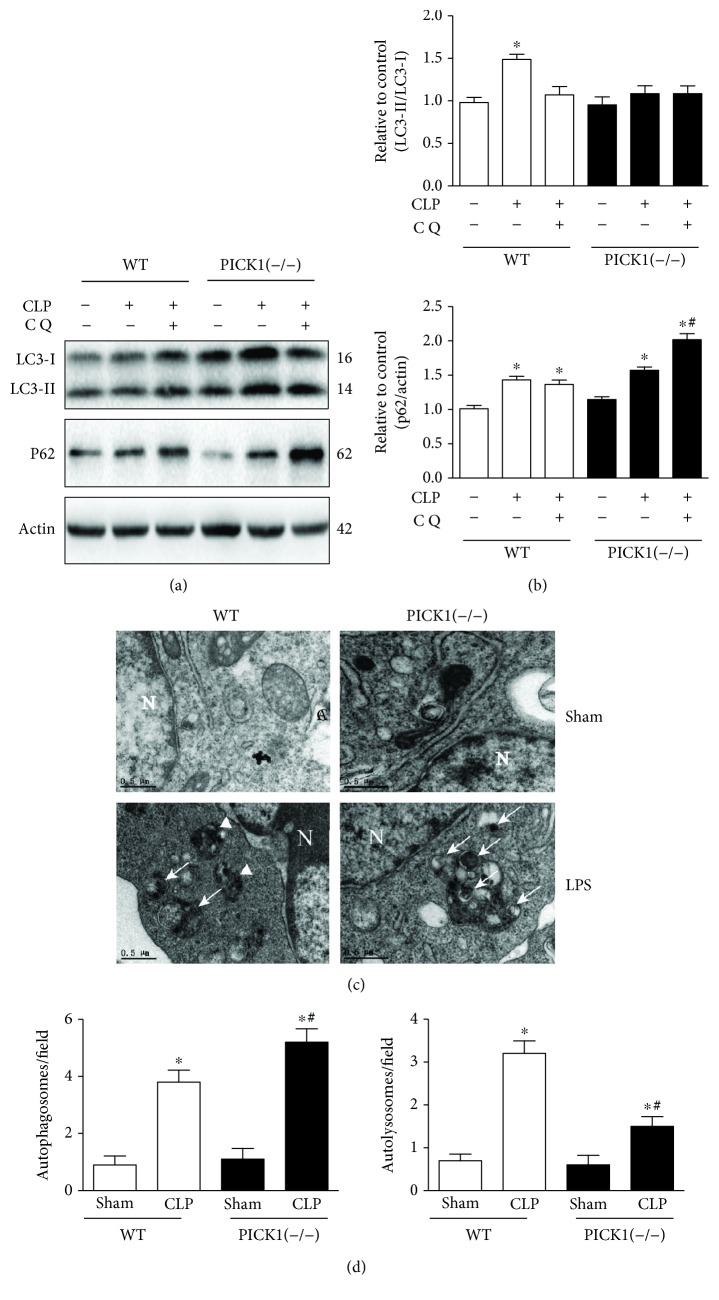
PICK1 deficiency disrupted the autophagic flux. Lungs were harvested 24 h post-CLP. (a, b) Representative Western blot images and statistical analysis of LC3-II/LC3-I ratio and p62 levels in *PICK1*^−/−^ mice. (c) Representative TEM images of autophagic structures in BMDMs. Autophagosomes are indicated by arrowheads and autolysosomes are shown by arrows (magnification, 10,500x). (d) Statistical analysis of autophagosomes and autolysosomes. Results represent the mean ± SEM of independent experiments of animals (*n* = 6) and cells (*n* = 3). ^∗^*P* < 0.05 vs. sham-operated group; ^#^*P* < 0.05 vs. CLP mice in the WT group.

To confirm the relationship between PICK1 and autophagy, we induced sepsis in PICK1^−/−^ mice. The conversion of LC3-I to LC3-II is the essential step in autophagosome formation; thus, we used the LC3-II/LC3-I ratio to observe the autophagic flux [23]. The results showed that LC3-II/LC3-I ratio was higher in the CLP group than that in the sham group in WT mice, but the ratio remained unchanged in PICK1^−/−^ mice post-CLP compared to that in the sham group. Additionally, p62 levels in the CLP group of PICK1^−/−^ mice made no statistical difference to those in the WT group. Therefore, we injected 60 mg/kg chloroquine intraperitoneally after CLP to block autophagosome–lysosome fusion for investigating the contribution of LC3-II/LC3-I ratio in PICK1^−/−^ mice. The results showed that chloroquine did not further increase LC3-II/LC3-I ratio but elevated p62 levels relative to the CLP group of PICK1^−/−^ mice, suggesting that the autophagic flux was disrupted in PICK1^−/−^ mice after CLP (Figures 2(a) and 2(b)). Macrophages often serve as the first barrier to confront invading pathogens [24], and autophagy in macrophages and immune function in septic mice are strongly related [25], then we detected the transmission electron microscopy (TEM) results in BMDMs to observation of autophagosomes and autophagosome–lysosomes. Autophagosomes are generally surrounded by double- or multimembrane structures engulfing cytoplasmic organelles and materials, such as mitochondria, whereas autophagosome–lysosome complexes contain digested components with a single membrane [26]. We found that more autophagosomes with double-membrane structures but less autolysosomes containing digested cytoplasmic components with single-membrane structures were detected in PICK1^−/−^ cells as compared with WT cells following LPS and IFN-*γ* treatment, indicating autophagosomes were accumulated in PICK1^−/−^ cells (Figures 2(c) and 2(d)). Together, these in vivo and in vitro results indicate that mice lacking PICK1 experienced disrupted autophagy progress after sepsis.

### 3.3. PICK1 Deficiency Amplifies the Damage Induced by Sepsis

**Figure 3:**
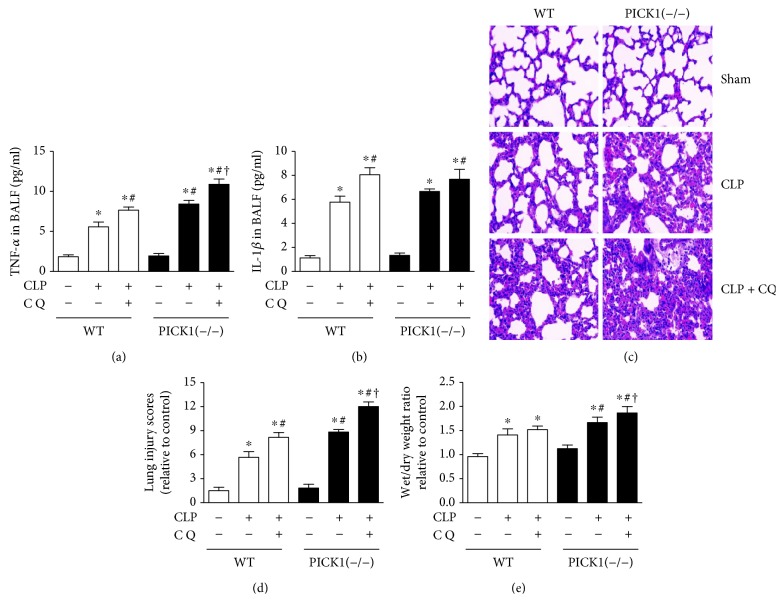
PICK1 deficiency leads to more serious damage. (a, b) Changes in TNF-*α* and IL-1*β* concentrations in BALF between groups. (c) Representative photomicrographs of lung sections stained with H&E at 24 h post-CLP in WT and *PICK1*^−/−^ mice (magnification, 200x). (d) Lung wet : dry weight ratio in WT and *PICK1*^−/−^ mice. Results represent the mean ± SEM (*n* = 6/group). ^∗^*P* < 0.05 vs. sham-operated group; ^#^*P* < 0.05 vs. CLP mice in the WT group; ^†^*P* < 0.05 vs. injection of chloroquine after CLP in the WT group.

As autophagic flux was disrupted in PICK1^−/−^ mice, we wondered whether it plays a protective or pernicious role in sepsis-induced ALI. The results showed that after injection of chloroquine, proinflammatory cytokine factors TNF-*α* and IL-1*β* were increased compared to the CLP group both in the WT and PICK1^−/−^ mice. However, the level of TNF-*α* in PICK1^−/−^ mice was higher than that in WT mice both in the CLP group and chloroquine group. There was no difference of IL-1*β* between WT and PICK1^−/−^ mice (Figures 3(a) and 3(b)). According to the morphologic evidence of hematoxylin and eosin staining (Figures 3(c) and 3(d)), we found that lung injury scores have higher level in PICK1^−/−^ mice than in WT mice after CLP with injection of chloroquine together or not, and the scores were highest in the chloroquine group. What is more, the wet/dry weight ratio (Figure 3(e)) showed the same results with H&E. Those results indicated that the lungs were more seriously damaged in PICK1^−/−^ as compared with those in WT mice after CLP. Moreover, chloroquine injection even worsened the damage relative to sham-operated mice of PICK1^−/−^ mice after CLP. We concluded that PICK1 deficiency inhibited the autophagic flux and amplify the damage in the sepsis-induced ALI. Since blocking of autophagy by chloroquine further increased lung injury, we speculate that increased autophagy plays a protective role in sepsis-induced lung injury.

### 3.4. PICK1 Deficiency Blocked Autophagic Flux via Impairing Lysosomes

**Figure 4:**
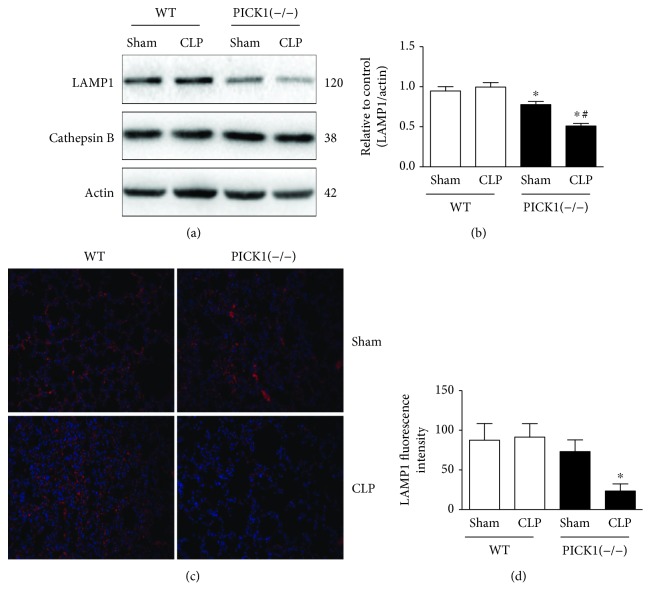
Lysosome is impaired in PICK1^−/−^ mice. (a, b) Lungs were harvested 24 h post-CLP. Representative Western blot images and statistical analysis of LAMP1 and cathepsin B in PICK1^−/−^ mice. (c, d) Immunofluorescence and statistical analysis of LAMP1 in *PICK1*^−/−^ mice post-CLP (magnification, 200x). Results represent the mean ± SEM (*n* = 6/group). ^∗^*P* < 0.05 vs. sham-operated group in WT mice; ^#^*P* < 0.05 vs. sham-operated group in PICK1^−/−^ mice.

Autophagy is a degradation process which depended on lysosomes [27]. Fusion of the autophagosome with a lysosome to form the autolysosome is a prerequisite for complete degradation of cargo content, which ensures proper disposal of damaged cellular biomolecules or organelles. Either lysosome damage or degradation process disrupted could cause autophagic flux blockage, leading to autophagosome accumulation and eventually autophagy dysfunction [28]. To elucidate the mechanism underlying how PICK1 deficiency inhibited autophagic flux, we assessed the lysosomal function through the expression of the lysosomal membrane proteins LAMP-1 with lysosomal protease protein cathepsin B (Figures 4(a) and 4(b)). As shown, LAMP-1 was significantly decreased in PICK1^−/−^ mice than that in WT mice after CLP. The expression was even lower than the sham-operated mice. But we did not find any changes in any group of cathepsin B. The immunofluorescence of LAMP1 also showed that there was a significant decline in PICK1^−/−^ mice after CLP. These results indicated that impairment of lysosome caused by PICK1 deficiency may account for the blockade of autophagic flux.

### 3.5. The mTOR Pathway Medicated by PICK1 Deficiency

**Figure 5:**
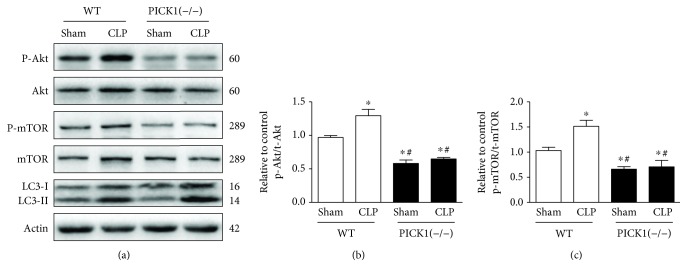
PICK1 participates in autophagy through dephosphorylation of Akt and mTOR. (a) Representative Western blot images and statistical analysis of p-Akt/t-Akt and p-mTOR/t-mTOR levels in *PICK1*^−/−^ mice. Results represent the mean ± SEM of independent experiments in mice (*n* = 4). ^∗^*P* < 0.05 vs. sham-operated group, ^#^*P* < 0.05 vs. CLP group.

There are many classical signaling pathways to regulate autophagy progress; among them, PI3K-Akt-mTOR pathway is a key survival-signaling pathway involved in modulating autophagy [29]. Besides, inhibition of mTOR in autophagic flux could not only be an autophagy induction but also a disruption [30]. It has also been reported that autophagy regulation could be an mTOR independent process. Therefore, we investigated relationships between PICK1 and the PI3K-Akt-mTOR signaling pathway associated with autophagy induction. We found that levels of phosphorylated p-Akt and p-mTOR, regulators of autophagy progress, increased at 24 h post-CLP relative to levels observed in the sham-operated group in WT mice. Whereas p-Akt and p-mTOR levels were significantly decreased in the sham-operated group and the CLP group in PICK1^−/−^ mice as compared with levels in WT mice (Figures 5(a)–5(c)). These data indicated that PICK1 participated in autophagy progression by regulating the PI3K-Akt-mTOR-signaling pathway.

## 4. Discussion

The main finding of this research is that PICK1 deficiency causes a disruption of autophagy progress and amplifies the damage in sepsis-induced ALI. Additionally, it is the impaired lysosomal function that accounts for the autophagosome accumulation. We also find that the PICK1-dependent autophagy is related to PI3K-Akt-mTOR-signaling pathway. These findings suggest a new concept of PICK1 associated with autophagy, and its deficiency may play a harmful role in sepsis.

Over the past two decades, the functions of PICK1 have been widely discussed in neurological disease and nonneurological disease. PICK1 was originally reported in neuros due to its interaction with protein kinase C (PKC). Through its distinctive structures, PICK1 interacts with broad ranges of neurotransmitter receptors, transporters, and enzymes to influence the synaptic function, resulting in neurological damage such as Parkinson's disease, epilepsy, and schizophrenia [31]. Besides, deficiency of PICK1 would cause male infertility in patients and animals as PICK1 is essential for vesicle trafficking from the Golgi apparatus to the acrosome in sperm cells [32]. What is more, PICK1 participates in the breast cancer though inhibiting TGF-*β* signaling thus initiating the early cancer [33]. Those reports indicated a strong relationship of PICK1 in oxidative stress, apoptosis, and inflammation. But the interrelation of PICK1 with autophagy is unclear. Our results showed that PICK1 deficiency in septic mice decreased the LC3-II/LC3-I ratio compared to the WT mice, and administration of autophagy inhibitor chloroquine on PICK1^−/−^ mice did not further increase the LC3-II/LC3-I ratio but the P62 level, indicating that the autophagosome accumulation was caused by autophagy dysfunction rather than autophagy activation. The TEM images in BMDMs also revealed that PICK1 deficiency increased autophagosome accumulation but reduced the autolysosomes, suggesting a disruption of autophagy. Our *in vitro* and *in vivo* results indicated that PICK1 is associated with autophagy, and its deficiency would block the autophagic flux. It is the first time to report the role of PICK1 with autophagy in sepsis-induced ALI.

Autophagy dysfunction is considered as the potential toxic mechanism of sepsis. Chung et al. reported that using autophagy inhibitor bafilomycin A1 significantly increased lipid accumulation and intensified the damage in endotoxin-induced liver injury [34]. Similarly, Pu et al. showed that loss of Atg7 enhanced inflammasome activation by increasing the production of IL-1*β* [35]. Suppression of autophagy in T cells accelerated apoptosis and increased mortality in sepsis model [36]. In recent years, studies have reported increased autophagy in lung disease resulting from exposure to stress agents, including hypoxia, oxidants, inflammation, ischemia–reperfusion, endoplasmic reticulum stress, pharmaceuticals, or inhaled xenobiotics. However, accumulating evidence revealed the protective effects of autophagy in acute lung dysfunction [1]. As the cytokine, H&E, and wet/dry weight ratio have shown in our study, lung damage was more serious in PICK1^−/−^ mice after CLP with disrupted autophagy. When autophagy inhibitor chloroquine was applied after CLP, the damage was even higher than the CLP group in PICK1^−/−^ mice. The results suggested that inhibition of autophagy caused by PICK1 deficiency was pernicious to lung injury, which was consistent with the previous researches.

Autophagy is a lysosome-dependent degradation process. Fusion of autophagosomes with lysosomes is necessary for complete autophagic flux [37]. Any failure of the steps in this process may lead to accumulation of toxic, structurally disruptive and damaged structures, and excessive cellular components. Therefore, the autophagy-lysosomal pathway (ALP) is an important mechanism for regulating homeostasis of intracellular environment. The destruction of lysosome comprises two sides named lysosomal membrane permeabilization (LMP) and lysosomal membrane rupture (LMR) [38]. Mutation of lysosome-associated membrane protein-1 (LAMP-1) and lysosome-associated membrane protein-2 (LAMP-2) influenced autophagosomes' combination with endosomal vesicles to lysosomes, and lysosomal proteases cathepsins are required for subsequent substrate degradation. Additionally, there are other genes and proteins that affect multiple steps of this progress. Rab7, a member of the small GTPase family, is also required for the fusion of autophagosomes and lysosomes in the sepsis-induced acute kidney injury [39]. Studies have also analyzed that a transcription factor EB (TFEB) is another regulator of lysosomes and autophagosomes, whose connection has not been clearly defined [40]. In PICK1^−/−^ mice, we found lower level of LAMP-1 than the WT mice after CLP, but we did not get any change on cathepsin B. These results suggested that PICK1 deficiency would destroy the structure of lysosome to influence the autophagic flux. As previously mentioned, the deficiency of PICK1 caused oxidative stress-dependent damage in mice [14]. Whether it is the excessive ROS production that destroys the function of lysosomes needs further dissection.

Activation of phosphatidylinositol 3-kinase (PI3K) and its downstream effectors Akt and mTOR are key survival modulators that promote cellular defense [41]. However, apart from the classical role of mTOR, it has also been reported that autophagy induction could be an mTOR-independent process. Tian et al. have reported that in a mice model of Parkinson's disease, acupuncture promoted the autophagic clearance of *α*-synuclein though regulating ALP proteins rather than mTOR-dependent pathway [42]. Furthermore, Manzoni et al. also found that activity of Leucine-rich repeat kinase 2 (LRRK2) negatively regulated macroautophagy independent of mTOR in astrocyte cell model [43]. Our results showed lower levels of p-Akt and p-mTOR as compared with WT mice after CLP, suggesting a role for PICK1 in modulating the PI3K-Akt-mTOR-signaling pathway. As PI3K-Akt-mTOR pathway is a classical pathway in which mTOR gets inhibited and leaded to enhanced autophagy induction [44], the decreased levels of p-Akt and p-mTOR were not corrected with the disrupted autophagic flux in PICK1^−/−^ mice. Interestingly, Cinà et al. have reported that in Podocytes, inhibition of mTOR could decrease autophagic lysosome reformation and accumulate autophagic vesicles, resulting in toxicity to the cell [45]. What is more, Rong et al. have also found that Drosophila spin mutants have influenced the reactivation of mTOR, leading to the lysosomal abnormalities [46]. The inhibition of Akt and mTOR pathway may affect the reformation of autolysosome and disrupt the autophagic flux in PICK1^−/−^ mice. The study offers a potential mechanism of PI3K-Akt-mTOR-signaling pathway with PICK1 and autophagic activity in the setting of sepsis.

## 5. Conclusions

**Figure 6:**
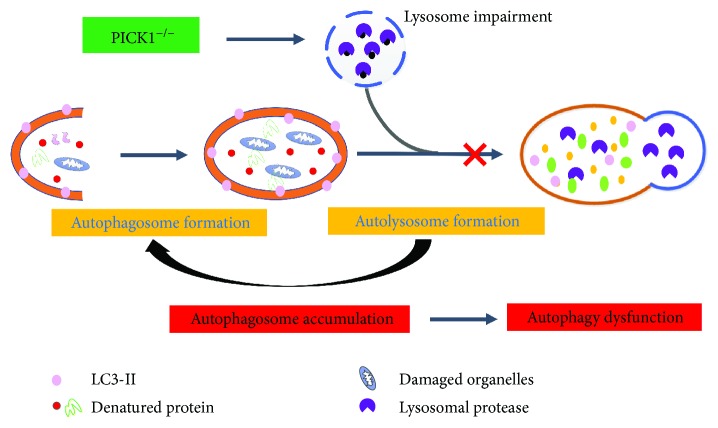
Proposed model for PICK1 deficiency-related autophagy in sepsis-induced acute lung injury. In PICK1^−/−^ mice, impaired lysosomal structure decreases the function of lysosomes and influences the formation of autophagosomes with it. Accumulation of autophagosomes eventually result in autophagy dysfunction.

In summary, we found that PICK1 deficiency disrupts the autophagic flux through impaired lysosomal function, which was harmful to the lung tissues (Figure 6). Additionally, the autophagy progress was relevant to PI3K-Akt-mTOR pathway. These results provide new insights into PICK1 function and might promote the development of therapeutic methods to treat sepsis-induced ALI.

## Data Availability

The data used to support the findings of this study are available from the corresponding author upon request.
